# Editorial: Highlights in diabetes clinical epidemiology, volume II

**DOI:** 10.3389/fcdhc.2025.1649441

**Published:** 2025-08-01

**Authors:** Georgia Vourli, Nikos Pantazis

**Affiliations:** ^1^ Georgia Vourli, Center for Research and Education in Public Health, Academy of Athens, Athens, Greece; ^2^ Department of Hygiene, Epidemiology and Medical Statistics School of Medicine National and Kapodistrian University of Athens, Athens, Greece

**Keywords:** novel diagnosis, epidemiology, care, adolescence, comorbidities

Diabetes mellitus continues to represent one of the most pressing global health challenges, not only because of its rising prevalence but also due to the growing complexity of its management and the expanding spectrum of associated comorbidities. Recent research published in *Frontiers in Clinical Diabetes and Healthcare: Volume II*, alongside the previously published Volume I Research Topic, offers valuable insights into the complex nature of diabetes, encompassing clinical phenotypes, diagnostic innovation, epidemiological trends, and emerging technological opportunities. In this editorial, we synthesize key findings from six recent articles that together reflect the evolving landscape of diabetes research and its implications for clinical practice and public health.

A prominent theme across these studies is the heterogeneity of diabetic manifestations and complications, particularly highlighted in the retrospective study by Almazrouei et al., which analyzed diabetic ketoacidosis (DKA) cases in adults in the United Arab Emirates (Almazrouei et al.). The study demonstrated that while DKA predominantly affects patients with type 1 diabetes (T1DM), those with type 2 diabetes (T2DM) present with significantly worse outcomes, including longer hospital stays and higher mortality. The results call attention to the clinical vigilance required, especially for T2DM patients at risk of DKA and the importance of tailored educational strategies addressing treatment adherence and infection control in both diabetic populations.

This theme of individualized risk continues in the work of Charkos and Getnet, who report a 53.2% prevalence of metabolic syndrome (MetS) among patients with T2DM in Ethiopia (Charkos and Getnet). This facility-based cross-sectional study identifies multiple modifiable factors, such as smoking, sedentary behavior, high BMI, and dietary patterns, that are strongly associated with MetS. These findings reinforce the urgent need for integrated non-pharmacological interventions, particularly in resource-constrained settings where the burden of cardiovascular complications is mounting.

In contrast to these clinical conditions, Jia and Yu shift the focus to the diagnostic frontier by evaluating assay technologies for population-based screening of T1DM (Jia and Yu). While the radio-binding assay (RBA) remains the current gold standard, the authors argue convincingly that its limitations in both cost-efficiency and disease specificity, render it unsuitable for large-scale preventive efforts. Multiplex platforms such as electrochemiluminescence (ECL) assays and 3-assay ELISA combining three IAbs, demonstrate improved predictive power and operational feasibility. This transition towards more refined, affinity-based diagnostics could mark a turning point in identifying preclinical T1DM, particularly among asymptomatic individuals who would otherwise remain undiagnosed until late-stage beta-cell failure.

The importance of early identification, which can now be assisted by novel methods as those previously mentioned, is echoed in the real-world cohort study by Weiner et al., which monitored adolescents with prediabetes (Weiner et al.). The study highlights two key points: First, the transition from prediabetes to type 2 diabetes (T2DM) in adolescents remains under-researched. It addresses that gap by leveraging a large dataset of electronic health records. Second, although this transition appears to be less frequent in adolescents compared to adults, the proportion of adolescents who do progress to T2DM is not negligible. These findings underscore the importance of continued vigilance, including regular monitoring and the implementation of preventive strategies.

In a complementary vein, the study by Li et al. delves into the psychosocial determinants of diabetes risk, revealing a nuanced interplay between sex, depression, sleep quality, and glycemic outcomes (Li et al.). Their results indicate that men are more susceptible to the diabetogenic effects of depression and poor sleep compared to women, suggesting a need to incorporate mental health screening into routine diabetes prevention strategies, particularly for male patients. This adds an important psychosocial layer to our understanding of disease etiology, bridging behavioral science with metabolic medicine.

Finally, Tura et al. present a critical appraisal of in-silico modeling for beta-cell function, raising pertinent questions about the clinical utility of computational approaches in endocrinology (Tura et al.). While the potential of such models for predicting insulin secretion and guiding therapy is acknowledged, the authors caution that widespread clinical adoption will require significant advances in model validation, integration with patient data, and clinician training.

Collectively, these six contributions reflect a broadening scope in diabetes research, from acute complications and comorbidities to long-term risk profiling, from biochemical markers to psychosocial dimensions, and from static observations to dynamic modeling. What emerges is a vision of diabetes care that is increasingly personalized, preventive, and data-driven, yet deeply contextualized within socioeconomic and behavioral realities.

For clinicians, these studies reinforce the importance of adopting a multidimensional approach to risk assessment that extends beyond glycemic control alone. For researchers, they highlight fertile ground for innovation in diagnostics, digital modeling, and lifestyle intervention trials. For public health policymakers, the evidence underscores the urgency of targeted prevention strategies across diverse populations, from adolescents at risk of T2DM to adults in under-resourced healthcare systems.

In conclusion, the fight against diabetes will not be won on a single front. Rather, it will require convergence: of technology with empathy, of science with community engagement, and of policy with person-centered care. The six studies reviewed herein mark important steps in that direction.

